# A Mixed-Methods Systematic Review on the Impacts and Implementation of Collaborative Electronic Documentation on Nurse-Patient Relationship

**DOI:** 10.1097/CIN.0000000000001263

**Published:** 2025-02-12

**Authors:** Gift Iwuchukwu, Minna Anttila, Tella Lantta, Jaakko Varpula, Maria Ameel

**Affiliations:** Department of Nursing Science, Faculty of Medicine, University of Turku, Finland (Ms Iwuchukwu and Drs Varpula, Ameel, Anttila, and Lantta); Centre for Forensic Behavioural Sciences, Swinburne University of Technology, Melbourne, Australia (Dr Lantta); and Department of Psychiatry Helsinki University Hospital and University of Helsinki, Finland (Dr Ameel)

**Keywords:** Collaborative documentation, Electronic health record, Mixed-methods systematic review, Nurse-patient relationship, Nursing documentation

## Abstract

The use of electronic health records challenges the nurse-patient relationship. Collaborative documentation could help to change this. The aim of this review was to provide a synthesis of current knowledge on the impacts, as well as barriers and facilitators of collaborative electronic documentation in nursing settings. A mixed-methods systematic review was conducted. The search was conducted in November 2022. The study used thematic analysis for qualitative data and descriptive analysis for quantitative data. Data integration was performed using a convergent integrated approach according to the Joanna Briggs Institute methodology. The methodological quality of the included studies was critically appraised using the Mixed-Methods Appraisal Tool. A total of 17 studies were included. Study types were qualitative (n = 10), quantitative (n = 2), and mixed methods (n = 5). Multiple implementation practices were identified in different types of nursing settings. The facilitators and barriers were related to characteristics of nurses, patients, technology, and organization. Education and organizational support were identified to be essential in successful implementation. Collaborative documentation could be a way to overcome the challenges in nursing process associated with electronic documentation, as it can save nurses' time and improve patient experience. Implementation needs to be carried out with end-user patients and nurses.

Patient involvement and access to health information are emphasized in health policies and health systems worldwide.^1,2^ Involvement enables patients to participate in decision-making regarding their care, take an active role, and collaborate with professionals toward health outcomes.^3^ Participation provides patients with relevant information about their health^4^ and improves patient satisfaction,^3^ patient empowerment^3,4^ and health literacy.^4,5^ Whereas patient involvement and collaboration have been emphasized in the care relationship, documentation is usually considered as a tool for use among clinicians. Legislation that requires patients to have access to their health record data challenges this assumption.^6^

Patient documentation has traditionally been carried out in paper format, but during recent decades, healthcare systems have adopted electronic health record (EHR) documentation for collecting and sharing patient information.^7^ The use of EHR makes it possible to gather large amounts of patient-related data to support clinical decision-making in real time, if the data are entered at the point of care.^8^

However, the impact of EHR on nursing processes has been partly negative. According to recent studies, nurses spend an average of 9%^9^ to 17%^10^ of their working time documenting care using the EHR. This is often viewed as time away from patients and considered burdensome by nurses.^11^ A recent review on the impact of EHR use on nurse-patient relationships concluded that using electronic documentation during patient encounters made the nurse-patient interaction more task-driven and inhibited communication.^12^ Collaborative electronic documentation (CED) together with the patient could be a way to change this. The EHR provides possibilities for patient collaboration in the documentation process. These processes include, for example, screen sharing, the use of patient portals, and contact channels within the EHR.^13^ For example, when sharing clinical notes with patients with mental health problems, positive stakeholder experiences have been reported,^4^ as well as success in eliciting patient feedback and input^14^ and reducing the time needed for documentation.^15^ More knowledge of evidence-based practices is required in order to incorporate CED in nursing practice and to better develop approaches toward CED.^14^

## AIM

The aim of this mixed-methods systematic review was to collect and provide a synthesis of current knowledge on the use of CED in nursing settings. We aimed to identify facilitators, barriers, and outcomes of CED and to provide knowledge on means for supporting CED implementation in clinical practice from both patients' and nurses' perspectives.

## METHODS

The Joanna Briggs Institute's (JBI's) mixed-methods synthesis approach was used to guide this study.^16^

The following research questions were addressed:

1. What is known of the practices for/of using CED in nurse-patient relationships?
2. What facilitators and barriers contribute to CED from nurses' and patients' perspectives?
3. What are the outcomes (benefits and disadvantages) of CED practices?

The review protocol was registered in the international database of prospectively registered systematic reviews, PROSPERO (CRD42022365360).

### Search Strategy

**FIGURE 1:**
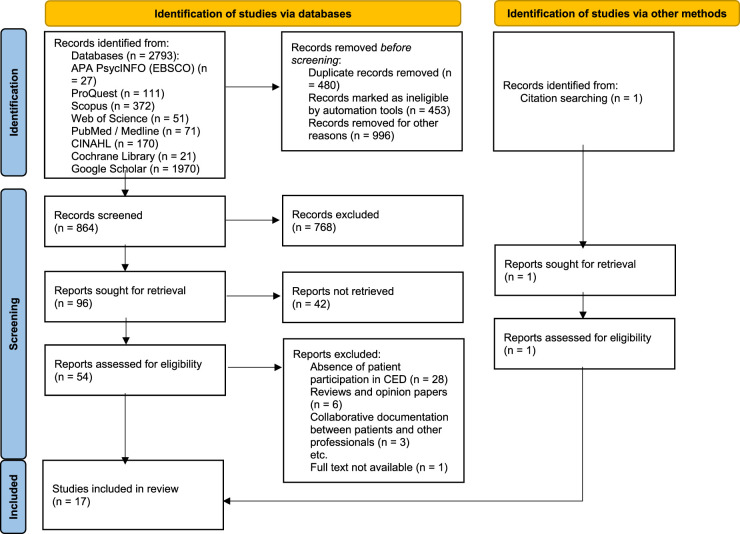
PRISMA 2020 flow diagram for this systematic review, which included searches of databases and other sources.

The search terms defining key elements of the review question included population (nurses, patients, family members), intervention (CED), comparison (none), outcome (facilitators, barriers of CED implementation benefits, disadvantages of CED practices), setting (all nursing settings) (PICOS) terms. Inclusion and exclusion criteria are described in detail in Table 1. Literature reviews were excluded.^17^ The terminologies, including keywords, free text words, truncations, Boolean operators, index, and Medical Subject Headings, were identified and compiled into comprehensive and specific search strings for the databases. The search strings were validated by an information specialist. The search strategy is presented in detail in Supplemental Table 1 (http://links.lww.com/CIN/A414). Because the study area is relatively new, the publication year of the articles used in the review was not restricted, thus providing a comprehensive overview of current literature. The databases used are described in Figure 1.

**Table 1:** Inclusion and Exclusion Criteria

Inclusion Criteria	Exclusion Criteria
**Population** Patients, nurses, consumers, service users, other professionals reported alongside nurses	**Population**Physicians, pharmacists
**Intervention** Collaborative documentation, electronic nursing documentation, CED	**Intervention**Joint electronic documentation between physicians and non-nurse professionals without patient participation
**Comparison/context** Nurse-patient relationship, nursing	**Comparison/context**Physician-patient, pharmacist-patient, psychiatrist-patient relationship
**Outcomes/study type** Support, experience, usability, acceptability, enhancement Primary research, quantitative, qualitative or mixed methods, case studies Full-text, peer-reviewed articles published in scientific journals in English	**Outcomes/study type**Books, study protocols, literature reviews, commentary, discussions, thesis papers, opinion papers, gray literature, studies not published in English

Titles and abstracts were blindly screened by two reviewers (anonymized), and full texts included were reviewed by two (anonymized). Reference lists of included studies were manually searched to identify any additional eligible studies. During the full-text screening, discussion between the three reviewers resolved inconsistencies pertaining to the relevancy for article inclusion. To ensure reproducibility and traceability, reporting followed PRISMA (Preferred Reporting Items for Systematic Reviews and Meta-Analyses).^18^

### Quality Assessment

The Mixed-Methods Appraisal Tool (MMAT) instrument was used to critically appraise the methodological quality of the studies used in this review.^19^ The instrument was developed for assessing the quality of systematic mixed studies comprising qualitative, quantitative, and mixed-methods studies. The MMAT comprises two sections, screening questions (n = 2) and methodological questions (n = 5), which access the methodological quality of the paper. Answers vary from yes (1), no (0), to “not applicable/can't tell” (N/A). Methodological questions are dependent on the study design: qualitative research, randomized controlled trials, nonrandomized studies, quantitative descriptive studies, or mixed-methods studies.^20^ Most of the studies had a high methodological quality ranking, with one exception. All studies were included in the review, as the MMAT appraisal instrument discourages the exclusion of studies with low methodological quality.^20^ Critical appraisal of the methodological quality of the studies was performed (Table 2) independently by two reviewers (anonymized) using the MMAT. Disparities identified from independent evaluations were discussed and resolved.

**Table 2:** Quality Assessment of Included Studies Using MMAT Tool

Author/Year	Screening Questions		Qualitative Research					Randomized Controlled Trials					Quantitative Descriptive Studies					Mixed-Methods Studies				
	S1	S2	1.1	1.2	1.3	1.4	1.5	2.1	2.2	2.3	2.4	2.5	4.1	4.2	4.3	4.4	4.5	5.1	5.2	5.3	5.4	5.5
Burkoski et al^21^ (2019)	1	1																0	0	0	0	0
De Groot et al^22^ (2021)	1	1	1	1	1	1	1															
De Groot et al^23^ (2022)	1	1	1	1	1	1	1															
Denneson et al^24^ (2017)	1	1	1	1	1	1	1															
Fernandes^25^ (2017)	1	1	1	1	1	1	1															
Galligioni et al^26^ (2015)	0	0																0	1	1	0	1
Gerber et al^27^ (2017)	1	1	1	1	1	1	1															
Glasper et al^28^ (2006)	1	1											1	1	1	1	0					
Graham et al^29^ (2018)	1	1	1	1	1	1	1															
Lezard and Deave^30^ (2021)	1	1	1	1	1	1	1															
Lindroth et al^31^ (2018)	1	1	1	1	0	1	1															
Lushin et al^32^ (2022)	1	1																1	1	1	1	1
McMath and Harvey^33^ (2004)	1	1																1	1	0	0	0
Misto et al^34^ (2019)	1	1																0	1	1	1	1
Pithara et al^35^ (2020)	1	1	1	1	1	1	1															
Rose et al^36^ (2014)	1	1	1	1	1	1	1															
Wang et al^37^ (2017)	1	1						1	1	1	0	1										

Note: MMAT items: 1 = yes; 0 = no; N/A = not applicable/can't tell; S1 = Are there clear research questions? S2 = Do the collected data allow to address the research questions? 1.1 = Is the qualitative approach appropriate to answer the research question? 1.2 = Are the qualitative data collection methods adequate to address the research question? 1.3 = Are the findings adequately derived from the data? 1.4 = Is the interpretation of results sufficiently substantiated by data? 1.5 = Is there coherence between qualitative data sources, collection, analysis and interpretation? 2.1 = Is randomization appropriately performed? 2.2 = Are the groups comparable at baseline? 2.3 = Are there complete outcome data? 2.4 = Are the outcome assessors blinded to the intervention provided? 2.5 = Did the participants adhere to the assigned intervention? 4.1 = Is the sampling strategy relevant to address the research question? 4.2 = Is the sample representative of the target population? 4.3 = Are the measurements appropriate? 4.4 = Is the risk of nonresponse bias low? 4.5 = Is the statistical analysis appropriate to answer the research question? 5.1 = Is there an adequate rationale for using a mixed-methods design to address the research question? 5.2 = Are the different components of the study effectively integrated to answer the research question? 5.3 = Are the outputs of the integration of qualitative and quantitative components adequately interpreted? 5.4 = Are divergences and inconsistencies between quantitative and qualitative results adequately addressed? 5.5. = Do the different components of the study adhere to the quality criteria of each tradition of the methods involved? Hong et al.^20^

### Data Extraction

Data extraction was guided by JBI's manual for evidence synthesis.^38^ The data extraction tool for qualitative research was JBI Qualitative Assessment and Review Instrument.^16^ For quantitative research, the following forms were used: JBI Qualitative Assessment and Review data extraction form for interpretative and critical research and JBI data extraction form for experimental/observational studies.^39^ These tools guided the data extraction and data relevant to the review questions. Data are organized into a table portraying the study characteristics and descriptive data (Table 3).

**Table 3:** Descriptive Characteristics and Other Descriptive Data Significant to the Study Objectives

Authors (Year) Country	Phenomena of Interest/Research Aim	Study Designs/Data Collections	Participants/Settings	CED Practice
Burkoski et al^21^ (2019), Canada	Impact of integrated bedside terminals (IBTs) on patient empowerment and nursing workflows	Mixed methods:surveys, interviews	n = 113 patientsn = 11 nursesHospital setting	The IBTs provide access to a range of convenience and entertainment services and access to personal health information
De Groot et al^22^ (2021), the Netherlands	Community nurses' experiences of patient participation in electronic nursing documentation, the challenges they face, and the strategies they use for dealing with challenges	Qualitative: interviews	n = 19 nursesHome care setting	Electronic nursing documentation
De Groot et al^23^ (2022), the Netherlands	Home-care patients perspective of participation in electronic nursing documentation	Qualitative: interviews	n = 21 patientsHome care setting	Patient is involved in the actual documentation in the electronic health record (EHR), and/or reviews, corrects and supplements the information documented
Denneson et al^24^ (2017), United States	Perspectives and experiences of mental health clinicians with OpenNotes to understand how they affect mental healthcare	Qualitative: interviews	n = 28 clinicians (psychiatrists, psychologists, social workers, nurse practitioners), nurses (RNs and licensed practical nurses)Medical center	Online patient portal through which patients can access their healthcare records and progress notes, refill prescriptions, and securely email their clinicians
Fernandes^25^ (2017), United States	Explore the hospitalized adult patient's experience during point-of-care electronic documentation	Qualitative: interviews	n = 20 patientsHospital setting	Clinical electronic documentation done at the point of care: the patient's bedside
Galligioni et al^26^ (2015), Italy	Describe the Safe Therapy Mobile system for the safe delivery of infusion chemotherapy in hospital wards and the Onco-TreC home monitoring system designed to increase patient/health professional interactions	Mixed methods: survey, testing, validation	n = 15 nursesn = 59 patientsHospital setting	A mobile phone or tablet diary app, which allows patients to record their state of health, the medications taken and side effectsWeb dashboard that allows health professionals to check the patient data and monitor toxicity and treatment adherence
Gerber et al^27^ (2017), United States	Identify nursing staff reactions to and perceptions of electronic portal use	Qualitative: interviews	n = 13 nursesOutpatient clinic	Electronic patient portal
Glasper et al^28^ (2006), United Kingdom	Inform the development of collaborative patient documentation	Quantitative: survey	n = 62 (medical consultants, nurses, sisters, ward managers, doctors, other)Hospital setting	Collaborative patient record system
Graham et al^29^ (2018), United States	Explore nurses' attitudes toward bedside documentation (BD) and to gain a better understanding of the practices in BD	Qualitative: interviews	n = 8 nursesHospital setting	BD in EHRs
Lezard and Deave^30^ (2021), United Kingdom	Explore what leads to inconsistency in EHR use	Qualitative:interviews	n = 12 nursesHome care setting	EHR use
Lindroth et al^31^ (2018), Sweden	How nurses' use data, gathered by patients with a mobile phone app, during consultations	Qualitative: observations, interviews, document analysis	Nurses and patientsHospital setting	Use of patient-generated datain consultations
Lushin et al^32^ (2022), United States	Feasibility and acceptability of collaborative documentation (CD) tool for implementing medication-assisted treatment	Mixed methods: interviews, surveys	n = 9 patients,n = 11 counselors (certified substance abuse counselors, licensed clinical social worker)Outpatient centers	A structured shared decision-making session guide for front-line counselors, with a health-record template using a CD approach. The session guide (CD MAT Tool) contains a training manual and session templates
McMath et al^33^ (2004), Scotland	Provide a method by which patients and professionals share information	Mixed methods: interview, survey	n = 3 patientsNurses, general practitioners, podiatrists, consultantsOutpatient setting	Patient-held record
Misto et al^34^ (2019), United States	Examine staff nurses' perception of the impact of electronic documentation in the presence of the patient on the nurse-patient relationship	Mixed methods: survey, interviews	n = 297 nurses Hospital setting	Electronic documentation in the presence of the patient
Pithara et al^35^ (2020), United Kingdom	Examine mental healthcare providers' views of and experiences with the care pathway tool (CPT) during the implementation and identify factors influencing implementation	Qualitative: interviews	n = 20 mental health providers (mental health support workers, peer support workers, psychiatrists, occupational therapists, community psychiatric nurses, social workers), managersOutpatient setting	An innovative mobile digital CPT to be used on a tablet computer
Rose et al^36^ (2014), United States	Describe the lived experience of patients communicating with nurse practitioners and physicians while using health records	Qualitative: interviews	n = 21 patientsMedical center	Use of paper health records and EHRs in the examination rooms
Wang et al^37^ (2017), China	Develop and evaluate a Web-based coaching program using EHRs	Randomized controlled trial: questionnaire, clinical variables (spirometer, dyspnea scale, walking test)	n = 130 patientsHospital	A Web-based coaching program using EHRs

### Data Analysis and Synthesis

Mixed-methods systematic review concepts and considerations were followed in the data analysis and synthesis: thus, (1) primary data were obtained from the studies; (2) quantitative data were transformed into qualitative format; (3) quantitative data were combined with qualitative data following transformation; (4) combined extracted data from qualitative studies (including data from the qualitative component of a mixed-methods study) resulted in the generation of qualitative evidence; and (5) allowed the quantitative and qualitative synthesis to occur simultaneously, a convergent integrated design was followed.^40^ “Qualitizing” data extracts from quantitative studies and converting it into “textual descriptions” allowed integration with qualitative data into a single mixed-methods synthesis, aimed to be confirmatory^40^ and to address the same research purposes and questions.^41^ The subtype used was a data-based convergent synthesis design, where qualitative and quantitative evidence is analyzed together using the same synthesis method (quantitative data are transformed into categories/themes), and the results are presented together.^42^

We used an inductive thematic analysis approach to analyze the integrated findings in accordance with Braun and Clarke,^43^ including familiarization of data, generation of codes, identifying, reviewing and naming themes, and presenting the final analysis embedded within an analytic narrative. Thematic synthesis is used when drawing conclusions on common elements across otherwise heterogeneous studies^16^ and different types of research evidence. The results are presented in tabulated matrices and in assimilative and summative narrative synthesis. An example of thematic analysis is given in Supplemental Table 2 (http://links.lww.com/CIN/A415).

The initial database search resulted in 2793 articles. After removal of 480 duplicates, 453 conference papers, books, and thesis papers and 996 inaccessible results from Google Scholar, the titles and abstracts of the remaining 864 articles were screened for eligibility. After excluding 768 articles, 96 articles remained for full-text screening. Of these, 42 papers comprised editorials, thesis papers, and abstract consortiums, leaving 54 papers, of which 38 were excluded as they comprised literature or scoping reviews or discussed CED in physician-patient or pharmacist-patient relationships. One study was included from manual searching of the reference list of the included studies. Seventeen studies met the inclusion criteria of the full-text review. The study selection is portrayed in the PRISMA flow diagram in Figure 1.

## RESULTS

### Study Characteristics and Types of CED

Characteristics of the included studies are summarized in Table 3. Included studies were published between 2004 and 2022. Of the 17 studies, 15 were published within the last 10 years. The majority of studies used qualitative designs (n = 10), five used mixed methods (n = 5), and two used quantitative designs. Studies were conducted in the United States (n = 7), United Kingdom (n = 3), the Netherlands (n = 2), Scotland, Sweden, Italy, China, and Canada (n = 1). Study participants included patients (n = 376) and nurses (n = 357) using various titles (ie, nurse practitioners, RNs, licensed practical nurses, community psychiatric nurses). Other specialist professionals mentioned alongside nurses in the same study were physicians (including psychiatrists, general practitioners), psychologists, social workers, consultants, counselors, podiatrists, support workers, occupational therapists, and organizational managers. Specialties varied from acute, primary, and childcare to oncology, substance use, and diabetes.

Five studies described the development, acceptability, feasibility, integration, or implementation of interventions for monitoring health, communicating, and documenting care with patients.^26,28,32,33,37^ Three studies discussed patients' perspectives and experiences of participating in the use of electronic documentation and strategies to enhance patient-provider relationships.^23,25,36^ Five studies examined nurses' perspectives of utilizing various electronic documentation practices in nursing care or in communication with patients.^22,27,29,30,35^ Three studies focused on the impact of electronic documentation on patient empowerment, patient care, and nurse-patient relationships,^21,24,34^ and one study focused on the influence of electronically generated patient health data in nurse-patient consultation.^31^ The most commonly used methods were interviews (n = 14) and surveys and questionnaires (n = 7). Three studies included both surveys and interviews. Other methods were observations (of clinical variables) and document analysis.

### Quality Assessment of the Included Studies

The quality appraisal for each study is summarized in Table 2. All 12 qualitative and quantitative studies were of high quality. Two of the five mixed-methods studies had high quality, two had moderate quality, and one had low quality.

### Practices for CED

The review identified several different types of CED practices (Table 3). These included shared use of the EHR by the nurse and the patient or family member for documentation at the point of care,^21,25,29^ collaboration and reviewing in the documentation,^22,23,27,30,34,36^ or use of patient-generated health data.^31,33^ In some cases, the EHR systems were aimed at documenting patient progress,^24^ preferences, mutually agreed treatment decisions and treatment plans,^32^ or introduced goal-oriented exercises for patients in accordance with the care plan.^35^ There was an application that allowed real-time communication between patients and healthcare professionals, while data were being documented in the hospital's information system.^26^ There were collaborative approaches that allowed participants across a variety of disciplines to use the patient record.^28,37^ Some applications were specifically used in home care settings.^22,23,26,30,31,33,34,36^ A common practice was that both nurses and patients documented directly into the system.^22,23,27,30,31,33,34,36^

### Facilitators and Barriers to CED

Four main themes and six subthemes were identified facilitating CED practices (Table 4). Four main themes and nine subthemes were identified as barriers to CED (Table 4). The main facilitator and barrier themes included nurses, patients, technology, and organization.

**Table 4:** Facilitators and Barriers of CED Use

CED Facilitators	
Main themes	Subthemes
Nurse-related facilitator	• Nurses' assistive and educative role • Familiarity with CED • Nurses style of communication
Patient-related facilitator	• Patient's interest and input
Technology-related facilitator	• Efficient and seamless device integration
Organizational facilitator	• Availability of structures, policies, directives, and incentives to adopt CED practices
CED Barriers	
Main themes	Subthemes
Nurse-related barriers	• Personal working method • Resistance to change • Disconnected communication
Patient-related barriers	• Patient passivity • Misuse of documentation channels • Factors related to patient age and illness
Technology-related barriers	• Challenges with device technique and connectivity problems • Documentation settings
Organizational barrier	• Lack of organizational support

### Facilitators

Nurse-related facilitator was nurses' assistive and educative role^21–23,25,26,34–36^ as professionals to use CED,^30,35^ to encourage patients in participating,^22,23,34–36^ and being aware of patient-associated factors influencing CED use.^21–23,35^ Such factors included patient's age, language specifications, technical literacy, education, and physical and psychological functional capacity. Familiarity with CED^30,32^ affected the nurses' fluency, ability and self-efficacy in using CED,^35,36^ and required exposure to and training in CED prior to implementation.^36^ Nurses' style of communication, that is, verbal, nonverbal, and eye contact^22,25,36^; obtaining patients' opinions^22,36^; and focusing on interaction^25,36^ were essential CED facilitators.

Patient-related facilitator was patients' interest and input,^21–23,31,32,35,36^ which helped them receive information about their health^22,23^ and provided patients with opportunities to correct nurses' documentation.^22–24^ Family caregivers' interest in nursing documentation was also relevant.^22^

Technology-related facilitator was efficient and seamless device integration^23,26,35^ regarding EHR, documentation devices, patient portals, and Web dashboards, which provided patients with opportunities to collaborate and to have control over individual care and care-related decisions.^23,35^

Organizational facilitator was availability of structures, policies, directives, and incentives to adopt CED^28,30,32,35,36^ together with standardized practices regarding objectives of documentation.^24,27–30^ In a study by Glasper et al,^28^ 94% of 62 professionals were of the opinion that the patient record should be accessible to all care providers, and 61% favored patient (and carer) contribution to documentation.

### Barriers

Nurse-related barriers were nurses' personal working methods^23,25^ when they failed to provide support for the patients^23^ and patients were unaware of documentation or what the nurses actually did when they were documenting.^21–23,25^ Resistance to change^21,24^ was another barrier. According to Denneson et al,^24^ nurses perceived an unequal distribution in power; that is, they had reduced control over information; hence, they were reluctant to grant patients autonomy over their health information. Another type of resistance to change included nurses' preference to use traditional documentation methods and practices such as paper documentation^22,34^ in the absence of patients. Nurses expressed prevalence of disconnected communication,^25,30,34,36^ indicating missed opportunities for contacting patients and maintaining eye contact,^25,29,34,36^ which they considered to hinder the nurse-patient communication.^24,29,30,34,36^

Patient-related barriers consisted of patient passivity^23^ if patients considered their care level insignificant, they had no personal interest in nursing documentation,^22,23,35^ or they did not want to burden their family caregiver by requesting their participation in the documentation.^22^ There were patients who misused documentation channels by overutilizing access, that is, by reporting emergencies or getting stuck in irrelevant information,^27^ increasing the volume of communication. Factors related to patient age and illness caused challenges, that is, old age,^21–23^ lack of physical well-being,^22,23^ psychoemotional state,^22,24,35^ concentration problems,^23^ or distrust in technology.^21,35^

Technology-related barriers in CED focused on challenges with device technique and connectivity problems.^21–23,25,35^ Patients had no required devices^22,23^ or lacked technological skills.^21–23,25^ Login problems,^35^ technical difficulties,^21–23,35^ or integration problems^23,30,35^ presented additional technology-related barriers. Documentation settings hindered CED practices.^22,23,25,29,34,36^ In some cases, patients mentioned that a spouse or another family caregiver stepped in,^23^ the place was messy or unhygienic,^30^ or there was lack of space.^30,34^

Organizational barrier was a lack of organizational support and inconsistencies in organizational approach,^27,29,35^ which led to disparities in CED practices.^24,30,35^ Another organizational barrier was the lack of resources and time for guidance and training.^24,29,35^

### The Outcomes (Benefits and Disadvantages) of CED

**FIGURE 2:**
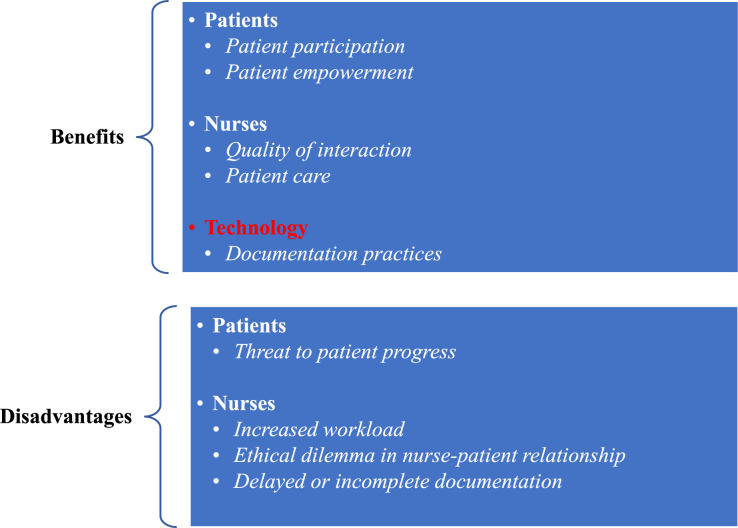
Outcomes of CED.

The review identified three main themes and six subthemes benefitting CED practices and two main themes and three subthemes that disadvantaged them. The main benefitting and disadvantaging themes related to patients and nurses. Additionally, benefits regarding work processes were identified (Figure 2).

### Benefits

Nurse related-benefits were that CED improved quality of interaction in nurse-patient relationships^21,24,31,33,34,36^ via communication^21,25,28,30–36^ and by enhancing caring relationships^21,24,25,29–34,36^ and mutual trust.^21,22,24,25,29–34,36^ CED improved patient care via information exchange.^21,25,26,28–31,33,34,36^ It facilitated care safety^25,26,28–30,36^ and improved transparency.^21,24,25,29–31,33,36^

Patient-related benefits were that it increased patient participation,^21–24,30–33,35,36^ encouraged patient engagement,^22,23,28,30–32,35,36^ and supported co-care^23–25,30–32,35,36^ and shared decision-making,^21–23,30–32,35,36^ especially in care planning^22,24^ and in understanding of overall health.^22,24,25,30–34,36,37^ CED increased patient empowerment,^21,24,33,36^ self-autonomy, and self-management,^21,24^ as patients were able to use their health information.^21,23–25,28,30–34,36,37^

Technology-related benefits were that it improved documentation practices^21,22,24,26,28,30,32–36^ and enhanced documentation quality by providing more accurate information^25,32,36^ that was documented in real time.^24–26,29–31,34,36^ It also provided clinicians with possibilities to reflect on their individual documentation practices.^24,30^

### Disadvantages

Nurse-related disadvantages included experiences of increased workload,^21,27,29,30,33,34^ feelings of burden^21,22,27,29,30,34^ regarding teaching and assisting patients with CED, and slowing down of work processes because of nonfunctional technological systems,^30^ leading to reduced prioritization of CED practices.^27,29,30,34^ The majority (60%) of 15 nurses thought that it reduced speed in performing nursing tasks.^26^ Another disadvantage was that nurses perceived CED to cause an ethical dilemma in nurse-patient relationships^24,29,34^ because of reduced focus on patients during documentation,^24,25,29,34^ resulting in reduced patient satisfaction.^24,29,34^ CED practices also resulted in delayed or incomplete documentation^22,24,30^ and complicated processes^27^ because of patient portal use, for example, if nurses had to safeguard test results from being released electronically to the patient prior to the office visit.

Patient-related disadvantages were that CED threatened patient progress when clinical documentation was inaccurate, progress notes left space for miscommunication or misinterpretation, and clinicians had to adjust their practices to protect patients and themselves from adverse consequences of CED. Such cases were encountered, for example, in mental health settings^24^ or if a patient was in a vulnerable situation, for example, domestic neglect.^22^

## DISCUSSION

Based on our search, this is the first review to describe the use of CED in nursing settings. Most of the 17 studies that met our inclusion criteria were published within the last 10 years, indicating the topicality of the study subject. The studies were mainly conducted using qualitative research methods for both nurses and patients. Nursing settings included homes and institutions with varying medical specialties.

The identified CED nurse-related facilitators and barriers aligned with previous research on the use of EHR during patient encounters with other clinical professionals.^12^ Nurses' professional way of using CED facilitated the use and improved patient care.^30,35^ Simultaneously, prejudices and nurses' personal way of working^23,25^ presented barriers to CED. Findings of this current review align with the ones in the integrative review by Forde-Johnston et al^12^ on the impacts of EHR on nurse-patient relationship. Both highlight nurses' role in practices in which EHR is used. Similarly, our findings support the suggestion to develop competencies for nurses on how to include patients and/or family members in the documentation process,^12^ and based on our findings, it is important to provide education on CED directly to patients.

This review included patients' and family members' views on CED usage, and the findings suggest that patients' interest and activity^21,23,31,32,35,36^ were important in the successful implementation of CED. The results of patient-related factors such as patient age and illness on CED use were mixed. For example, in a study on integrated bedside terminals, Burkoski et al^21^ found no statistically significant differences between patient age and CED users versus nonusers, whereas qualitative study did suggest a difference in the use between generations. The functionality of technology also had an impact on CED use. These challenges could be tackled by including end-users, both nurses and patients, in the planning process.^44^

Increased workload,^21,27,29,30,33,34^ ethical questions,^24,29,34^ and incomplete documentation^24,30^ were common experiences at the very beginning and could even threaten patient progress before CED use became daily practice. This suggests a need for strong organizational support for improving CED practices, especially during the implementation phase.^24,27,29,30,35^ Organizational support requires commitment, preparedness for the technology and CED, and engagement to implement practices in daily clinical care. Similarly to research with other clinical staff members,^15^ to de Groot et al,^22^ patient participation in the documentation can ultimately save nurses' time, in comparison to extensive hours spent documenting postconsultation and provide greater possibilities for mutually shared decision-making.

It is also important to note that negative impacts on patients were reported in instances where bedside documentation occurred without a clear CED practice such as visualization of the EHR together with the patient,^29,32^ suggesting that CED could be a way to overcome problems identified with bedside documentation.

At the same time, more understanding on the effects of possible barriers to CED including the lack of sufficient language skills on patients' side and strategies to overcome these is needed. The need to make the EHR accessible to patients is supported by legislation in many countries by requiring patients the right to read their healthcare information.^6^ There are differences between countries on the levels of openly accessible information (eg, whether nursing notes are included) as well as possibilities to partially delay accessibility of texts for patients,^6^ but it seems evident that open notes are the future, and CED could be a way to introduce health records to patients.

Additionally, different types of nursing settings can challenge the implementation of CED in different ways. Our review covered studies from several different nursing settings, but due to the small number of studies per setting, we are unable to draw conclusions on differences between these. Yet, this needs more research as the ways of CED in different types of settings differ and can include various practices from the use of remote patient portals to screen sharing. Previous research has shown that collaboration is easier to implement when the EHR documentation structure supports the clinical workflow.^45^ We believe that in order to achieve this, nurses and patients should be seen as end-users of the EHR and participate in the design of platforms and implementation.

There are also other positive experiences concerning technology use. In a study by Wang et al,^37^ CED had a positive impact on patients' physiological symptoms. However, system usability requires modifications preventing the system from becoming cumbersome, fragmented, and complex, which reduces the enthusiasm of new users and disadvantages patients who struggle to find relevant information, possibly resulting in them never returning to the platform.^46^ The challenge for nursing is to value technology and still maintain caring.^29^ Supporting patients to participate in CED could be a way to foster the nurse-patient relationship and the active participation of patients in their care process, in the era of electronic documentation.

### Study Strengths and Limitations

The strength of this review is that it identified both positive and negative experiences of a variety of CED practices with different patient groups as well as facilitators and barriers. Moreover, several authors were involved in order to reduce bias in the screening process, data selection, extraction, and analysis, and this process ensured the adequacy and reliability of the selected papers. We feel confident that the results of this review provide generalizable knowledge about the study topic.

Even though a systematic search strategy was applied in this review, there are some limitations. Searches were limited to articles published in English, which may have resulted in the exclusion of relevant articles from the review. The search relied on a selected set of keywords, and although all efforts were taken to include relevant search terms, it is possible that some relevant studies were omitted. There were alternative concepts, terms were used interchangeably, there was some variability in the systems and methods, and sometimes articles were poorly described. Therefore, the articles were carefully analyzed to determine their relevance to the study aim. Google Scholar was used as a gray literature database source to maximize the coverage of our searches. But, we failed to retrieve almost 1000 references from the database due to technical issues. However, we do not believe that these limitations weaken the findings of the review, because it only concerned Google Scholar references, and even hand search was conducted. The research field is rapidly growing, indicating the necessity for frequent review updates.

## CONCLUSION

This mixed-methods systematic review is the first to synthesize research on the use of CED in nursing settings. Nurses are often the first point of contact in providing direct care to patients. Findings indicate that there are experiences of facilitators of and barriers to CED use in daily practice.

Based on this review, CED could be a way to overcome the challenges identified with EHR use in nursing settings. More research is needed to examine the impact of CED on best practices in daily patient care. Further research should focus on the design and implementation of practices supporting organizational actions toward the use of CED. For example, end-user involvement to ensure the feasibility in the clinical workflow in the implementation process is essential in future attempts to implement CED practices.

## RELEVANCE TO CLINICAL PRACTICE

As the number of people, healthcare staff, patients, and family members using technology increases in healthcare, the need for collaboration in electronic documentation is likely to increase. Patients have increasing access to their EHR both internally within the clinic as well as remotely using apps and other online channels. Nurses need to understand this change and embrace their central role in this change, and organizations need to support the inclusion of CED in everyday practices. This includes the need to remove structural barriers and improve implementation strategies such as education and training to support CED use. The development of software and hardware technology needs to be conducted in cooperation with end-users including nurses, patients, and family members. It is necessary to build capacity for a sustainable workforce in nursing care that fosters high-quality care, patient involvement, and user satisfaction.

## Supplementary Material

## References

[1] European Patients' Forum . EPF at OECD: focus on patient involvement and patient safety. 2022. https://www.eu-patient.eu/news/latest-epf-news/2019/epf-at-oecd-focus-on-patient-involvement-and-patient-safety/

[2] Organisation for Economic Co-operation and Development . Health for the people, by the people: building people-centred health systems. 2021. https://www.oecd.org/health/health-for-the-people-by-the-people-c259e79a-en.htm

[3] Tambuyzer E Pieters G Van Audenhove C . Patient involvement in mental health care: one size does not fit all. Health Expectations. 2014;17(1): 138–150. doi:10.1111/j.1369-7625.2011.00743.x. 22070468 PMC5060706

[4] Schwarz J Bärkås A Blease C . Sharing clinical notes and electronic health records with people affected by mental health conditions: scoping review. JMIR Mental Health. 2021;8(12): e34170. doi:10.2196/34170. 34904956 PMC8715358

[5] World Health Organization . Patient engagement: technical series on safer primary care.; 2016. https://apps.who.int/iris/bitstream/handle/10665/252269/9789241511629-eng.pdf;sequence=1

[6] Hägglund M McMillan B Whittaker R Blease C . Patient empowerment through online access to health records. BMJ. 2022;378: e071531. doi:10.1136/bmj-2022-071531. 36175012 PMC9518004

[7] O'Donnell HC Suresh S, Council on Clinical Information Technology . Electronic documentation in pediatrics: the rationale and functionality requirements. Pediatrics. 2020;146(1): 0. doi:10.1542/peds.2020-1684. 32601127

[8] Hardiker NR Dowding D Dykes PC Sermeus W . Reinterpreting the nursing record for an electronic context. International Journal of Medical Informatics. 2019;127: 120–126. doi:10.1016/j.ijmedinf.2019.04.021. 31128823

[9] Bingham G Tong E Poole S Ross P Dooley M . A longitudinal time and motion study quantifying how implementation of an electronic medical record influences hospital nurses' care delivery. International Journal of Medical Informatics. 2021;153: 104537. doi:10.1016/j.ijmedinf.2021.104537. 34343955

[10] Khan AR Rosenthal CD Ternes K Sing RF Sachdev G . Time spent by intensive care unit nurses on the electronic health record. Critical Care Nurse. 2022;42(5): 44–50. doi:10.4037/ccn2022518. 36180057

[11] Olivares Bøgeskov B Grimshaw-Aagaard S . Essential task or meaningless burden? Nurses' perceptions of the value of documentation. Nordic Journal of Nursing Research. 2018;39(1): 9–19.

[12] Forde-Johnston C Butcher D Aveyard H . An integrative review exploring the impact of electronic health records (EHR) on the quality of nurse-patient interactions and communication. Journal of Advanced Nursing. 2023;79(1): 48–67. doi:10.1111/jan.15484. 36345050 PMC10100205

[13] Milne H Huby G Buckingham S . Does sharing the electronic health record in the consultation enhance patient involvement? A mixed-methods study using multichannel video recording and in-depth interviews in primary care. Health Expectations. 2016;19(3): 602–616. doi:10.1111/hex.12320. 25523361 PMC5055250

[14] Matthews EB . Computer use in mental health treatment: understanding collaborative documentation and its effect on the therapeutic alliance. Psychotherapy (Chic). 2020;57(2): 119–128. doi:10.1037/pst0000254. 31599638

[15] Matthews EB Peral M . Using collaborative documentation to support person-centered care in substance use settings. The Journal of Behavioral Health Services & Research. 2024;51(1): 74–89. doi:10.1007/s11414-023-09866-z. 37907671

[16] Lockwood G Porritt K Munn Z . Chapter 2: systematic reviews of qualitative evidence. In: Aromataris E Munn Z , eds. JBI Manual for Evidence Synthesis. 2020: https://synthesismanual.jbi.global .

[17] Pearson A White H Bath-Hextall F Salmond S Apostolo J Kirkpatrick P . A mixed-methods approach to systematic reviews. International Journal of Evidence-Based Healthcare. 2015;13(3): 121–131. doi:10.1097/XEB.0000000000000052. 26196082

[18] Page MJ McKenzie JE Bossuyt PM . The PRISMA 2020 statement: an updated guideline for reporting systematic reviews. Systematic Reviews. 2021;10(1): 89. doi:10.1186/s13643-021-01626-4. 33781348 PMC8008539

[19] Hong QN Pluye P Fàbregues S . Improving the content validity of the Mixed Methods Appraisal Tool: a modified e-Delphi study. Journal of Clinical Epidemiology. 2019;111: 49–59.e1. doi:10.1016/j.jclinepi.2019.03.008. 30905698

[20] Hong QN Fàbregues S Bartlett G . The Mixed Methods Appraisal Tool (MMAT) version 2018 for information professionals and researchers. Education for Information. 2018;34(4): 285–291. doi:10.3233/EFI-180221.

[21] Burkoski V Yoon J Hall TNT . Patient empowerment and nursing clinical workflows enhanced by integrated bedside terminals. Nursing Leadership (Toronto, Ontario). 2019;32(SP): 42–57. doi:10.12927/cjnl.2019.25815. 31099746

[22] De Groot K Sneep EB Paans W Francke AL . Patient participation in electronic nursing documentation: an interview study among community nurses. BMC Nursing. 2021;20(1): 72. doi:10.1186/s12912-021-00590-7. 33933079 PMC8088564

[23] De Groot K Douma J Paans W Francke AL . Patient participation in electronic nursing documentation: an interview study among home-care patients. Health Expectations. 2022;25(4): 1508–1516. doi:10.1111/hex.13492. 35384167 PMC9327866

[24] Denneson LM Cromer R Williams HB Pisciotta M Dobscha SK . A qualitative analysis of how online access to mental health notes is changing clinician perceptions of power and the therapeutic relationship. Journal of Medical Internet Research. 2017;19(6): e208. doi:10.2196/jmir.6915. 28615152 PMC5489707

[25] Fernandes A . Patients' experiences, expectations, and satisfaction with point-of-care electronic documentation. Journal of Informatics Nursing. 2017;2(4): 6–18.

[26] Galligioni E Piras EM Galvagni M . Integrating mHealth in oncology: experience in the province of Trento. Journal of Medical Internet Research. 2015;17(5): e114. doi:10.2196/jmir.3743. 25972226 PMC4468599

[27] Gerber DE Beg MS Duncan T Gill M Craddock Lee SJ . Oncology nursing perceptions of patient electronic portal use: a qualitative analysis. Oncology Nursing Forum. 2017;44(2): 165–170. doi:10.1188/17.ONF.165-170. 28222081 PMC7066859

[28] Glasper EA Holmes CW Brown KL Newton J . Shared records: towards collaborative working with families. Pediatric Nursing. 2006;18(1): 34–37. 16518952

[29] Graham HL Nussdorfer D Beal R . Nurse attitudes related to accepting electronic health records and bedside documentation. CIN: Computers, Informatics, Nursing. 2018;36(11): 515–520. doi:10.1097/CIN.0000000000000491. 30399004

[30] Lezard R Deave T . The factors influencing community nurses' usage of electronic health records: findings from focus groups. British Journal of Community Nursing. 2021;26(12): 604–610. doi:10.12968/bjcn.2021.26.12.604. 34878908

[31] Lindroth T Islind AS Steineck G Lundin J . From narratives to numbers: data work and patient-generated health data in consultations. Studies in Health Technology and Informatics. 2018;247: 491–495. 29678009

[32] Lushin V Matthews E Stanhope V . Feasibility and acceptability of collaborative documentation tool for implementing medication-assisted treatment in substance use disorder counseling. Journal of Social Work Practice in the Addictions. 2022;23(3): https://www.tandfonline.com/doi/abs/10.1080/1533256X.2022.2040115 .

[33] McMath E Harvey C . Complex wounds: a partnership approach to patient documentation. British Journal of Nursing. 2004;13(11): S12–S16. doi:10.12968/bjon.2004.13.Sup2.13234. 15218440

[34] Misto K Padula C Bryand E Nadeau K . Nurses' perception of the impact of electronic documentation on the nurse-patient relationship. J Nurs Care Qual. 2019;34(2): 163–168. doi:10.1097/NCQ.0000000000000339. 29975218

[35] Pithara C Farr M Sullivan SA . Implementing a digital tool to support shared care planning in community-based mental health services: qualitative evaluation. Journal of Medical Internet Research. 2020;22(3): e14868. doi:10.2196/14868. 32191210 PMC7118546

[36] Rose D Richter LT Kapustin J . Patient experiences with electronic medical records: lessons learned. Journal of the American Association of Nurse Practitioners. 2014;26(12): 674–680. doi:10.1002/2327-6924.12170. 25234112 PMC4307644

[37] Wang L He L Tao Y . Evaluating a Web-based coaching program using electronic health records for patients with chronic obstructive pulmonary disease in China: randomized controlled trial. Journal of Medical Internet Research. 2017;19(7): e264. doi:10.2196/jmir.6743. 28733270 PMC5544894

[38] Aromataris E Munn Z , eds. JBI Manual for Evidence Synthesis. JBI; 2020. https://synthesismanual.jbi.global . 10.46658/JBIMES-20-01

[39] Pearson A Field J Jordan Z . Appendix 3: data extraction tools. In: Evidence-Based Clinical Practice in Nursing and Health Care. United Kingdom: John Wiley & Sons, Ltd; 2006:183–186. doi:10.1002/9781444316544.app3

[40] Lizarondo L Stern C Carrier J . Chapter 8: mixed methods systematic reviews. In: Aromataris E Munn Z , eds. JBI Manual for Evidence Synthesis. 2020: https://synthesismanual.jbi.global .

[41] Sandelowski M Voils CI Barroso J . Defining and designing mixed research synthesis studies. Research Scholar. 2006;13(1): 29. PMC2809982 20098638

[42] Hong QN Pluye P Bujold M Wassef M . Convergent and sequential synthesis designs: implications for conducting and reporting systematic reviews of qualitative and quantitative evidence. Systematic Reviews. 2017;6(1): 61. doi:10.1186/s13643-017-0454-2. 28335799 PMC5364694

[43] Braun V Clarke V . Using thematic analysis in psychology. Qualitative Research in Psychology. 2006;3(2): 77–101. doi:10.1191/1478088706qp063oa.

[44] Martikainen S Kaipio J Lääveri T . End-user participation in health information systems (HIS) development: physicians' and nurses' experiences. International Journal of Medical Informatics. 2020;137: 104117. doi:10.1016/j.ijmedinf.2020.104117. 32179254

[45] Matthews EB . Integrating the electronic health record into behavioral health encounters: strategies, barriers, and implications for practice. Administration and Policy in Mental Health. 2017;44(4): 512–523. doi:10.1007/s10488-015-0676-3. 26208693

[46] Lyles CR Nelson EC Frampton S Dykes PC Cemballi AG Sarkar U . Using electronic health record portals to improve patient engagement: research priorities and best practices. Annals of Internal Medicine. 2020;172(11 suppl): S123–S129. doi:10.7326/M19-0876. 32479176 PMC7800164

