# Supplementary material for: A Mixed-Methods Systematic Review on the Impacts and Implementation of Collaborative Electronic Documentation on Nurse-Patient Relationship
Source: Comput Inform Nurs. 2025 Feb 12;43(12):e01263. doi: 10.1097/CIN.0000000000001263 (PMC12704668; doi:10.1097/CIN.0000000000001263)
Supplement: Supplementary file 1 [file nxn-43-e01263-s001.docx]

Supplementary file 1. Search terms used in database search

| **Databases searched** | **Search terms** |
| --- | --- |
| APA PsycINFO (EBSCO) | Search date: 24.11.2022  (consumer* OR patient* OR client OR "service user*") AND (nurs* OR nurse*) AND ("collaborative documentation*" OR "cooperative documentation*" OR "joint documentation*" OR "shared documentation*" OR "electronic documentation*" OR "electronic nursing documentation*" OR "electronic health record*" OR "electronic medical record*" OR "electronic patient record*" OR "documentation*" OR "concurrent documentation*" OR "bedside documentation*" OR "information system*") AND ("nurse-patient relationship*" OR "client-therapist relationship*" OR "client-provider relationship*" OR "nurse-patient communication*" OR "nurse-patient interaction*" OR "therapeutic relationship*" OR "therapeutic alliance*" OR "patient-provider teamwork*")  Limited by: Scholarly (Peer Reviewed) Journals; English; Document Type: Journal Article; Exclude Dissertations; Exclude Book Reviews; Exclude Non-Article Content |
| ProQuest | Search date: 23.11.2022  noft(patient* OR clients* OR consumer* OR "service user" OR "service users") AND noft(nurse* OR nurs*) AND noft("collaborative documentation*" OR "cooperative documentation*" OR "joint documentation*" OR "shared documentation*" OR "electronic documentation" OR "electronic nursing records*" OR "electronic nursing documentation*" OR "electronic health record*" OR "electronic medical record*" OR "electronic patient records*" OR "documentation*" OR "concurrent documentation*" OR "bedside documentation*" OR "information system") AND noft("nurse-patient relationship*" OR "client-provider relationship*" OR "nurse-patient communication*" OR "nurse-patient interaction*" OR "therapeutic relationship" OR "therapeutic alliance" OR "patient-provider teamwork*")  Limited by: Peer reviewed, English |
| Scopus | Search date: 24.11.2022  (TITLE-ABS-KEY (consumer* OR patient* OR client* OR "service user*" ) AND TITLE-ABS-KEY (nurs* OR nurse*) AND TITLE-ABS-KEY ("collaborative documentation*" OR "cooperative documentation*" OR "joint documentation*" OR "shared documentation*" OR "electronic documentation*" OR "electronic nursing documentation*" OR "electronic health record*" OR "electronic medical record*" OR "electronic patient record*" OR "documentation*" OR "concurrent documentation*" OR "bedside documentation*" OR "information system*") AND TITLE-ABS-KEY ("nurse-patient relationship*" OR "client-therapist relationship*" OR "client-provider relationship*" OR "nurse-patient communication*" OR "nurse-patient interaction*" OR "therapeutic relationship*" OR "therapeutic alliance*" OR "patient-provider teamwork*" ) ) AND ( LIMIT-TO ( DOCTYPE , "ar" ) OR LIMIT-TO ( DOCTYPE , "re" ) ) AND ( LIMIT-TO ( LANGUAGE , "English" ) ) AND ( LIMIT-TO ( SRCTYPE , "j" ) ) |
| Web of Science | Search date: 24.11.2022  (((TS=(patient* OR clients* OR consumer* OR "service user*" )) AND TS=(nurs* or nurse*)) AND TS=("collaborative documentation*" OR "cooperative documentation*" OR "joint documentation*" OR "shared documentation*" OR "electronic documentation*" OR "electronic nursing records*" OR "electronic nursing documentation*" OR "electronic health record*" OR "electronic medical record*" OR "electronic patient records*" OR "documentation*" OR "concurrent documentation*" OR "bedside documentation*" OR "information system*" )) AND TS=("nurse-patient relationship*" OR "client-provider relationship*" OR "nurse-patient communication*" OR "nurse-patient interaction*" OR "therapeutic relationship*" OR "therapeutic alliance*" OR "patient-provider teamwork*" ) |
| PubMed / Medline | Search date: 24.11.2022  ((consumer* or patient* or client* or "service user*") and (nurs* or nurse*) and ("collaborative documentation*" or "cooperative documentation*" or "joint documentation*" or "shared documentation*" or "electronic documentation*" or "electronic nursing documentation*" or "electronic health record*" or "electronic medical record*" or "electronic patient record*" or " documentation*" or "concurrent documentation*" or "bedside documentation*" or "information system*") and ("nurse-patient relationship*" or "client-therapist relationship*" or "client-provider relationship*" or "nurse-patient communication*" or "nurse-patient interaction*" or "therapeutic relationship*" or "therapeutic alliance*" or "patient-provider teamwork*")).mp. [mp=title, book title, abstract, original title, name of substance word, subject heading word, floating sub-heading word, keyword heading word, organism supplementary concept word, protocol supplementary concept word, rare disease supplementary concept word, unique identifier, synonyms] |
| CINAHL | Search date: 22.11.2022  (patient* OR clients* OR consumer* OR "service user*" ) AND ( nurse* OR nurs*) AND ("collaborative documentation*" OR "cooperative documentation*" OR "joint documentation*" OR "shared documentation*" OR "electronic documentation*" OR "electronic nursing records*" OR "electronic nursing documentation*" OR "electronic health record*" OR "electronic medical record*" OR "electronic patient records*" OR "documentation*" OR "concurrent documentation*" OR "bedside documentation*" OR "information system*") AND ("nurse-patient relationship*" OR "client-provider relationship*" OR "nurse-patient communication*" OR "nurse-patient interaction*" OR "therapeutic relationship*" OR "therapeutic alliance*" OR "patient-provider teamwork*")  Limited by: Abstract Available; English Language; Peer Reviewed |
| Cochrane library | Search date: 22.11.2022  (consumer* OR patient* OR client* OR service NEXT user*) AND (nurs* OR nurse*) AND (collaborative NEXT documentation* OR cooperative NEXT documentation* OR joint NEXT documentation* OR shared NEXT documentation* OR electronic NEXT documentation* OR electronic NEXT nursing NEXT documentation* OR electronic NEXT health NEXT record* OR electronic NEXT medical NEXT record* OR electronic NEXT patient NEXT record* OR documentation* OR concurrent NEXT documentation* OR bedside NEXT documentation* OR information NEXT system*) AND (nurse-patient NEXT relationship* OR client-therapist NEXT relationship* OR client-provider NEXT relationship* OR nurse-patient NEXT communication* OR nurse-patient NEXT interaction* OR therapeutic NEXT relationship* OR therapeutic NEXT alliance* OR patient-provider NEXT teamwork*) |
| Google Scholar | Search date: 26.11.2022  consumer* OR patient* OR client* OR "service user*" AND nurs* OR nurse* AND "collaborative documentation*" OR "cooperative documentation*" OR "joint documentation*" OR "shared documentation*" OR "electronic documentation*" OR "electronic nursing documentation*" OR "electronic health record*" OR "electronic medical record*" OR "electronic patient record*" OR "documentation*" OR "concurrent documentation*" OR "bedside documentation*" OR "information system*" AND ("nurse-patient relationship*" OR "client-therapist relationship*" OR "client-provider relationship*" OR "nurse-patient communication*" OR "nurse-patient interaction*" OR "therapeutic relationship*" OR "therapeutic alliance*" OR "patient-provider teamwork*" |
