# Supplementary material for: A Mixed-Methods Systematic Review on the Impacts and Implementation of Collaborative Electronic Documentation on Nurse-Patient Relationship
Source: Comput Inform Nurs. 2025 Feb 12;43(12):e01263. doi: 10.1097/CIN.0000000000001263 (PMC12704668; doi:10.1097/CIN.0000000000001263)
Supplement: Supplementary file 2 [file nxn-43-e01263-s002.docx]

Supplementary Table 1 Example of thematic analysis.

| Facilitators | Main theme | Sub themes | Citation quotes | References | Total  reference  counts, n |
| --- | --- | --- | --- | --- | --- |
|  | Nurse-related facilitator | *Nurses’ assistive and educative role*  (Nurses awareness of their own role and help patients to participate in the documentation/ confidence to introduce CED system features to users/Self-efficacy in nurses- fluent ability to use tool) | *“Nurses should tailor their encouragement of patient participation to individual patients' needs and abilities. Furthermore, they should be aware of their own role and help patients to participate in the documentation.”* | De Groot et al. 2022, Fernandes 2017, Graham et al. 2018, Pithara et al. 2020, Rose et al. 2017 | 5 |
|  |  | *Familiarity with CED tool (*Piloting practice, engaging nurses as recruiters and trainers, aids possibility for constant system improvements, Ongoing training during implementation) | “Involving peers on the ground to act as recruiters and trainers” | Galligioni et al. 2015, Lushin et al. 2022, Pithara et al. 2020, | 3 |
|  |  | *Patient consultation and focused interaction during CED* | “During both types of interactions, the nurse can incorporate verbal and non-verbal cues such as smiling, body positioning that is inclusive of the patient, and intermittent eye contact to demonstrate that the nurse is engaged and attentive” | De Groot et al. 2022, Fernades A.2007, Pithara et al. 2020 | 3 |
|  |  | *Awareness of user specific needs (*Age factor, specific user needs e.g. language, ability to engage with technology) | “Younger or more highly educated patients who received technical nursing care tended to fall in the type ‘high need, high ability’, whereas most elderly patients or less educated patients who received personal nursing care were assigned to the type ‘low need, low ability’.” | Burkoski et al. 2019, De Groot et al. 2022, Pithara et al.2020 | 3 |
|  |  | *Awareness of benefits of CED practices to patients* (Positive attitude towards technology use /  identifying patient benefits in the use of CED practices/knowledge of practices) | “recognising patient benefit was a prime motivator in clinicians’ use of and engagement with new technologies.” | Graham et al. 2018,  Lezard and Deave 2021, Pithara et al. 2020 | 3 |
|  | Patient-related facilitator | *Patient interest, patient* (Opportunities for patient input, perspective, patient inclusion) | “stated some kind of personal interest as a reason for their need to participate. According to these patients, participation provided them with relevant information about their health situation and gave them insights into the nurses' assessment of their health situation.” | De Groot et al. 2022, Lindroth et al. 2018, Rose et al. 2017 | 3 |
